# Health-system equity, egalitarian democracy and COVID-19 outcomes: An empirical analysis

**DOI:** 10.1177/1403494820982106

**Published:** 2021-01-09

**Authors:** Krishna Chaitanya Vadlamannati, Arusha Cooray, Indra de Soysa

**Affiliations:** 1 School of Politics and International Relations (SPIRe), University College Dublin (UCD), Ireland; 2 Former Ambassador of Sri Lanka to Norway, United Nations University, Finland; 3 Center for Poverty Analysis (CEPA), Sri Lanka; 4 Institute for Sociology and Political Science, Norwegian University of Science and Technology (NTNU), Norway

**Keywords:** Egalitarian governance, COVID-19, health system capacity, neoliberal pandemics

## Abstract

**Aims:**

The COVID-19 pandemic has led to a spate of studies showing a close connection between inequitable access to health care, welfare services and adverse outcomes from the pandemic. Others have argued that democratic governments have generally failed relative to more autocratic ones, simply because autocrats can make the hard choices required for stemming the spread of viruses. We address this question by asking whether more ‘egalitarian’ forms of democracy matter, given that they contain more equitable health-care access *and* societal infrastructure, such as social capital and trust.

**Methods:**

We use standard regression techniques, including instrumental variables analysis addressing endogeneity on COVID-19 testing and deaths data as of the end of May and beginning of September. We use novel data from the Varieties of Democracy Project on health-system equity and egalitarian democracy.

**Results:**

Our results suggest that more equitable access to health care increases testing rates and lowers the death rate from COVID-19. Broader egalitarian governance, measured as egalitarian democracy, however, shows the opposite effect. Thus, factors associated with health-care capacity to reach and treat matter more than broader societal factors associated with social capital and trust. The results are robust to alternative testing procedures, including instrumental variable technique for addressing potential endogeneity.

**Conclusions:**

Despite a great deal of public health focus on how equitable governance helps fight the adverse effects of so-called neoliberal pandemics, we find that broadly egalitarian factors have had the opposite effect on fighting COVID-19, especially when an equitable health system has been taken into account. Fighting disease, thus, might be more about the capacity of health systems rather than societal factors, such as trust in government and social capital.

## Introduction

The celebrated economic historian, Barry Eichengreen, suggests that the black–white disparity in COVID-19-related deaths in the USA can be traced directly to differences in welfare policies, which in turn can be blamed on racism and societal injustice [1]. His analysis is based on the well-established claims about the weakness of welfare states when ethnic differences are high and social capital and trust are low [2,3]. Jeffrey Sachs writes:

High inequality undermines social cohesion, erodes public trust, and deepens political polarization, all of which negatively affect governments’ ability and readiness to respond to crises. This explains why the United States, Brazil, and Mexico account for nearly half of the world’s reported deaths since the start of the pandemic. [4]

Indeed, a number of celebrated public-health scholars argue that the lack of inclusive, pro-poor governance is at the heart of the spread of many epidemics, such as obesity, drug abuse and even homicide [5–7]. These so called neoliberal pandemics are blamed directly on policies favouring capital and markets at the expense of community health and welfare [8]. Apparently, existing societal inequity, including health inequalities, exacerbate the unequal effects of COVID-19 in what some call a syndemic pandemic [9]. These observations prompt the question as to whether an ‘egalitarian democracy’,^1^ which contains greater equality in the distribution of political power resources, has greater inclusivity in political decisions and provides broadbased access to public goods, including health, generates favourable outcomes regarding the COVID-19 pandemic. Naturally, more egalitarian governance contains more equitable health systems, with greater capacity for reaching and treating people, thus stemming the spread of the virus. Using data on COVID-19 testing and death rates, we examine to what extent COVID-19-related outcomes might be explained by health-system ‘capacity’ compared to broadly egalitarian social and political governance. We also assess how an accessible health system conditions specific pandemic-targeting policy, such as testing policy and the stringency of lockdown, on COVID-related deaths.

For illustrative purposes, consider the examples of Taiwan and Sweden. Taiwan is hardly a Scandinavian-style democracy but has a capable health system where all citizens, and foreign residents (for at least six months), are entitled to a government insurance plan. Thus, an equitable and capable health system perhaps explains the country’s success in containing the virus, not necessarily broad-based equity. Similarly, Australia has a relatively equitable health-care system, even if a Scandinavian-style welfare state is absent [10]. Australia has experienced a lower death rate than some other advanced countries with more egalitarian governance. The idea that equality of access to health care reduces the impact of epidemics and pandemics is highly intuitive. A well-functioning health-care system, where the poor have access to health care on a par with the rich, is likely to have high capacity in terms of reaching and treating people, thereby cauterising the spread of a virus and minimising mortality. Nevertheless, many of these countries also adopted ‘emergency’ rules and ‘extraordinary’ measures that targeted the spread of the pandemic. These additional measures are independent of access to the health-care system or any other notion of broad-based egalitarian processes.

In the case of Sweden, it was argued that broad societal trust and social capital would be a critical factor in controlling the virus without much need for extraordinary measures [11]. Sweden’s strategy of ‘lockdown light’ was formulated on the basis of mutual trust between citizens and between citizens and the state, where people are urged to use their own judgement and voluntarily follow directives without strict government enforcement of lockdown. Apparently, Scandinavian-style welfare states can afford to fight neoliberal pandemics due to state–society dynamics associated with a strong welfare state and high social capital [9]. Such egalitarian values and infrastructure allegedly help collective outcomes because of shared values of community. Rather than administrative capacity alone, broad-based elgalitarian governance strengthens social capacities, which also seemingly builds resilience against disease.

The Swedish expectation, however, has not been met. When taken as a proportion of each country’s population, the numbers show that Sweden had 10.3% infections and 0.06% deaths compared to 0.023% infections and 0.002% deaths from COVID-19 in Norway. Thus, Sweden shows a death rate 30 times greater than Norway’s. Similarly, Finland, Iceland and Denmark also show much smaller death rates compared to Sweden.^2^ The equality of access to health care, however, is very similar across these countries, as are broad welfare policies and democratic inclusivity, which supposedly lead to high social capital and political trust. This comparison might indicate that health-care equity matters for fighting disease not because of the broader societal implications of societal trust in an egalitarian democracy, but rather because access to health care simply captures organisational capacities of health-care systems^3^ to deal more effectively with a pandemic. The governments of Vietnam, New Zealand and South Korea invested heavily in critical health-care facilities, and perhaps, as a result, had the capability to respond effectively to the COVID-19 crisis purely from the perspective of health-system capacity rather than the broad societal equity associated with strong welfare states [12]. Compared to Norway’s stricter lockdown, Sweden’s strategy of reliance on social capacity thus seems to have fared less well, despite having very similar health-care system capacities as the countries mentioned above.

Equality and justice are goods in their own right, and they are usually identified with democracies. However, not all democracies are the same [13,14]. How democracies respond to health crises relative to other regimes is not that clear. For example, the tough choices required to be made by public-health experts for fighting disease may clash with the competing priorities of ordinary people. If Swedish public-health experts could rely on the citizenry to trust their judgement, the same could not be said for many other industrialised democracies, such as the USA where some armed citizens have even stormed government buildings demanding an end to lockdown. Populist leaders, such as President Jair Bolsanaro in Brazil and Vladimir Putin in Russia, delayed their response to the virus for reasons of electoral popularity. Indeed, many less democratic regimes have been quite successful at curbing the coronavirus (e.g. China, Sri Lanka and Vietnam) compared to some full democratic regimes (e.g. the USA, the UK, Spain and Italy), while democracies with robust health-care systems have been able to deal with the virus more effectively (e.g. Germany, Australia and New Zealand). Could it be that these democracies have succeeded due to their broadly egalitarian governance rather than health-system capacity alone? After accounting for the capacity of the health-care system, it is not clear whether there are additional benefits to fighting disease from the broader setting of egalitarian governance, which economists, such as Jeffrey Sachs, and many public-health scholars hail as the antidote to a ‘syndemic pandemic’. From the observations above, we thus derive the following hypotheses to be tested empirically:

*Hypothesis 1: Equality in access to health care reduces the societal impact of health pandemics.* ^4^
*Hypothesis 2: Health-care equity should matter more than broad egalitarian governance for reducing the harm from health pandemics.*

## Data and methods

### Model specifications

We utilised a cross-section of data for 210 countries (see Supplemental Table A1 for the list of countries). COVID-19 testing and death rates were measured on 25 May 2020. The correlation between the May data and data for 25 June is almost identical at *r*=0.96, suggesting that the cross-sectional variation remained steady over a month of measurement. We also tested the data accumulated up to the month of September. We estimated the following equation:

(1) $$\ln{\left(\mathrm{COVID}\right)}_{c}=\varphi_{c}+\beta HCE_{c}+\beta Z_{c}+\lambda_{r}+\omega_{c}$$

where ln(*COVID*)_*c*_ captures COVID-19 tests per million (log) and COVID-19 deaths per million (log) in country *c* as of 25 May 2020.^5^ The Worldometers data are real-time data that are also the main source for the Coronavirus Government Response Tracker maintained by Oxford University and utilised by several others [15,16].^6^ *HCE_c_* measures the extent of equity in health care in country *c*. The Varieties of Democracy (V-Dem) project measures the degree to which any given country at any given point in time provides access to adequate health care for the poor that is comparable to the health care accessed by the rich. The V-Dem egalitarian democracy index includes several aspects of equity that measure the equality in distribution of political power in any given society in terms of gaining access to government and to resources that empower people politically and enable all people to participate meaningfully [14,17]. The V-Dem data on equity are generated by asking several country experts to score countries on the following question ‘To what extent is high-quality basic health care guaranteed to all, sufficient to enable them to exercise their basic rights as adult citizens?’, according to the scale in Table I.

**Table I.** Response scale for the question ‘To what extent is high-quality basic health care guaranteed to all, sufficient to enable them to exercise their basic rights as adult citizens?’.

**0**: Extreme. Provision of high-quality basic health care is extremely unequal, and at least 75% of citizens receive such low-quality health care that it undermines their ability to exercise their basic rights as adult citizens. **1**: Unequal. Provision of high-quality basic health care is extremely unequal and at least 25% of citizens receive such low-quality health care that it undermines their ability to exercise their basic rights as adult citizens. **2**: Somewhat equal. Basic health care is relatively equal in quality, but 10–25% of citizens receive such low-quality health care that it undermines their ability to exercise their basic rights as adult citizens. **3**: Relatively equal. Basic health care is overall equal in quality, but 5–10% of citizens receive such low-quality health care that it probably undermines their ability to exercise their basic rights as adult citizens. **4**: Equal. Basic health care is equal in quality and <5% of citizens receive such low-quality health care that it probably undermines their ability to exercise their basic rights as adult citizens.

The expert codings are subject to rigorous scrutiny and testing using item response theory that reduces uncertainty and assigns a single value to each country for each year. The ordinal coding is then transformed to be an interval scale indicator suitable for analysis across countries. Equality of access to health shows a strong correspondence with the World Bank’s World Development Indicators (WDI) data on infant mortality rate (*r*=−0.75) and a measure of government health-care expenditure as a share of gross domestic product (*r*=0.69), as well as with the Global Burden of Disease project’s indicator of health access and quality index (*r*=0.84).^7^

Our second main variable of interest was V-Dem’s egalitarian democracy index. An egalitarian democracy builds on the theorised notion that individuals from all social groups ought to be equally capable of exercising their political rights and freedoms and of influencing political and governing processes. Underlying this broad principle are two main sub-components: equal protection and equal distribution of resources and income protection (stronger equity). Equal protection implies that the state grants and protects rights and freedoms evenly across social groups [14]. They argue that greater egalitarian processes make a democratic polity more effective. Equality among groups would produce lower levels of polarisation and help resolve political and policy disputes more effectively than less egalitarian democratic processes [3,14]. The index of egalitarian democracy related only moderately with equitable access to health care, where one explains roughly 65% of the variance of the other.

Additionally, we tested the conditional effects of two government policy stances towards fighting the COVID-19 pandemic with our two main variables of interest on the outcome measured as deaths per million. The first of these two broad policy stances, government testing policy, is an index developed by Oxford University researchers [15]. The index captures the extent to which testing is available freely to asymptomatic people. The second policy stance is the stringency of lockdown, which captures variation in containment and closure policies of governments as of 25 May 2020. The index is a composite measure consisting of seven different response indicators: school and workplace closures, cancellation of public events, restrictions on the size of public gatherings, closure of public transport, internal movement restrictions, international travel restrictions and public information campaigns [16]. These conditional effects should tell us more about how health-system equity and egalitarian governance matter for fighting COVID-19.

The vector of control variables (***Z_c_***) included other potential determinants of COVID-19 outcomes that might be related with our main variables of interest. We included the level of development measured as per capita income in 2010 US dollar constant prices obtained from the World Bank (2019). Income level has a bearing on COVID-19 tests and deaths via its impact on health-care equity, as richer countries should have greater demand for social equity and have higher infrastructural capacity. Next, we included a measure of urbanisation (percentage share of urban population), as studies show that the transmission of COVID-19 is higher in urban centres because of the ease of transmission and contraction due to travel, connection to outside world and so on, and urbanisation relates to the nature of egalitarian processes associated with modernisation [18]. Finally, we included a measure of the share of the population aged >65 years in country ***c*** sourced from the WDI data platform because research shows that the fatality rate from COVID-19 rises sharply with age due to co-morbidities [19]. We use the past five-year average on each of these variables. The descriptive statistics are provided in Supplemental Table A3, and the details on definitions and data sources are provided in Supplemental Table A4. We limited the controls in order to avoid over-fitting the data. We estimated ordinary least square (OLS) specifications that include Huber–White corrected standard errors robust to heteroskedasticity. We added geographic regional dummies (**λ_*r*_**) to account for regional heterogeneity which may hide time invariant local-level factors, such as climate, geographic distances and cultural practices that influence the spread of disease.

### Endogeneity issues

It is plausible that health-care equity is an outcome rather than cause of poor health, or if both outcome and the independent variable were explained by some unmeasured higher-order variable. This issue is not trivial, since those who argue that health-care equity affects how the system responds to health pandemics also make causal claims [20,21]. To address the problem of endogeneity, we used a two-stage least-squares instrumental variable (2SLS-IV) estimator, using the number of years since independence as our instrument. The longer a country has been independent, the less likely it is to reverse historic inequities inherited at the time of independence. This feeds into the institutional persistence mechanism highlighted by many scholars who suggest that weak institutions inherited at the time of independence become irreversible, as they tend to persist and endure over time [22,23]. The duration of independence, however, should have no systematic bearing on how many COVID-19 tests and deaths a country has incurred, since viruses do not follow colonial history. The validity of the instrument depends on two conditions. The first is instrument relevance – that is, the selected instrument must be correlated with the explanatory variable in question, otherwise it has no power. Several experts on the topic suggest examining the joint *F*-statistic on the excluded instrument in the first-stage regression and the Kleibergen–Paap *F*-statistic [24]. The second condition is that the selected instrument should not differ systematically with the error term in the second stage of the equation – that is, (*ω_it_*|*IV_it_*)=0. It should not have any direct effect on the outcome variable of interest – COVID-19 tests and deaths – except through the institutional channel. Our instrument satisfies these conditions, as noted by the *F*-test and Kleibergen–Paap *F*-statistic.

## Results

Table II reports the impact of equity in health care on COVID-19 tests and deaths. Columns 1 and 2 show the results estimated with OLS using basic control variables and controlling for geographic regional dummies. Columns 3 and 4 present findings using the 2SLS-IV estimator. Columns 5 and 6 capture estimations based on COVID-19 tests and deaths as of 7 September 2020 (the latest data before submission).

**Table II.** The relative effects of health-care equity and egalitarian democracy on COVID-19 tests and deaths per million (log).

	(1)	(2)	(3)	(4)	(5)	(6)
	Tests	Deaths	Tests	Deaths	Tests	Deaths
Health-care equity	0.557***	−0.321**	0.650**	−1.361***	0.479*	−1.477***
	(0.136)	(0.140)	(0.318)	(0.434)	(0.278)	(0.387)
Democracy index	−1.162	2.176***	−1.356	4.545***	−1.069	2.819**
	(0.751)	(0.805)	(0.963)	(1.357)	(0.858)	(1.215)
Per capita GDP (log)	0.465***	0.519***	0.431**	0.927***	0.484***	0.806***
	(0.165)	(0.171)	(0.189)	(0.240)	(0.158)	(0.245)
Urban population share	0.0144*	0.00957	0.0138*	0.0146	0.0157**	0.0250***
	(0.00806)	(0.00890)	(0.00799)	(0.0105)	(0.00618)	(0.00954)
Population share 65 years old	−0.00676	0.0139	−0.0102	0.0353	−0.0321	0.0327
	(0.0375)	(0.0358)	(0.0360)	(0.0421)	(0.0328)	(0.0463)
Constant	4.701***	−3.616***	5.058***	−7.631***	6.263***	−4.472*
	(1.145)	(1.303)	(1.513)	(2.073)	(1.229)	(2.321)
Estimator	OLS	OLS	2SLS-IV	2SLS-IV	2SLS-IV	2SLS-IV
Regional fixed effects	Yes	Yes	Yes	Yes	Yes	Yes
First-stage *F*-statistics			21.97***	24.60***	23.26***	24.82***
Cragg–Donald Wald *F*-statistic			16.64***	21.13***	16.26***	17.25***
Kleibergen–Paap Wald *F*-statistic			16.72***	19.17***	17.53***	18.20***
No. of countries	152	151	152	151	161	167
*R* ^2^	0.705	0.624	0.703	0.493	0.670	0.295
*First-stage regressions*						
Years since independence			−1.211***	−1.265***	−1.223***	−1.234***
			(0.258)	(0.255)	(0.253)	(0.247)
*Control variables*			Yes	Yes	Yes	Yes
Regional fixed effects			Yes	Yes	Yes	Yes
No. of countries			152	151	161	167

Standard errors in parentheses. Source: Authors’ compilation based on estimation.

*** *p*<0.01; **p<0.05; *p<0.1.

OLS: ordinary least squares; 2SLS-IV: two-stage least-squares instrumental variable.

As seen there, equal access to health care has a positive impact on COVID-19 tests, which is significantly different from zero at the 1% level. Furthermore, column 2 shows that equity in health-care access has a negative effect on COVID-19 deaths, which is statistically significant at the 5% level. Interestingly, egalitarian democracy is negative on tests and positive on deaths at conventional levels of statistical significance. These results are robust across the columns in Table II. Broad egalitarian governance, once the health system is controlled, has negative effects on fighting pandemics. These results support both hypotheses stated above.

The substantive effects are large. A standard deviation increase above the mean value of health-care access yields a 1.31% increase in COVID-19 tests per million (log), which is roughly two-thirds the standard deviation of our dependent variable. A standard deviation increase above the mean value of the health-care equity index is associated with a 0.38% decrease in COVID-19 deaths per million (log), which is roughly 20% of the standard deviation of the dependent variable. Similarly, a standard deviation increase of egalitarian democracy above the mean reduces COVID-19 tests by 15% of a standard deviation of COVID-19 testing and 26% of a standard deviation of the death rate. These results are substantively quite large.

With respect to controls, both per capita income and urban population share show positive effects on tests. Interestingly, while the effect of income on COVID-19 deaths is positive, the effect of urbanisation, especially on deaths, remains statistically not significant. These results are intuitive, as richer countries have had higher exposure. It seems that the greater egalitarian values and processes contained within egalitarian democracies do not distinguish them from other democracies, since some studies have found that higher democracy measured in standard ways is also associated with higher COVID-19 deaths [25]. Notice that the effect of urbanisation on COVID-19 tests remains positive and significantly different from zero at the 10% level. We also do not find any statistical correlation between COVID-19 outcomes and age structure. Our results suggest that equality in access to health care matters more than broad egalitarian governance for reducing the harm from health epidemics because access to health care most likely increases the capacity to deal with them. It seems that broader forms of equity captured by egalitarian democracy reduce a state’s effectiveness against COVID.

In columns 3–6, we present the results with instrumental variable (IV) estimations. Notice that the results in columns 5 and 6 are estimated with the newly released 7 September data on COVID-19 tests (column 5) and deaths (column 6). While columns 3 and 5 report the results of COVID-19 tests, columns 4–6 capture COVID-19 deaths. There are three observations to be drawn from these results. First, the IV estimation results of health-care equity on COVID-19 tests per million in columns 3–5 and deaths per million in columns 4–6 are similar to those reported in our baseline estimates in columns 1 and 2. Second, as seen from columns 3–6, not only are the effects of health equity statistically significant, but the impact is large. Third, notice that the additional statistics provided in columns 3–6 in Table II suggest that the selected instrument is valid. The joint *F*-statistic from the first stage rejects the null that the instrument selected is not relevant. In fact, we obtained a higher joint *F-*statistic and a Kleibergen–Paap statistic on both estimation models reported in column 3, which remains significantly different from zero at the 1% level. Taken together, our results on the impact of equity in health-care access remain robust to alternative estimation techniques and endogeneity concerns. The results of the control variables are roughly the same as those reported in columns 1 and 2.

In Table III, we introduce interaction terms between health-care equity and measures capturing specific government actions – namely, testing policy and stringency of policy aimed at COVID-19. Columns 1 and 2 show the conditional effect of health-care equity and government testing policy and health-care equity and the stringency index on COVID-19 deaths per million. Columns 3 and 4 report the interaction effects for egalitarian democracy, testing policy and the stringency index respectively on COVID-19 deaths per million. It should be noted that neither of these policy measures alone have any statistically significant effect on the COVID-19 outcomes tested above.

**Table III.** Conditional effects of health-care equity and government policies and egalitarian democracy and government policies on COVID-19 deaths.

	(1)	(2)	(3)	(4)
	Tests	Deaths	Deaths	Deaths
Health-care equity×COVID-19 testing policy	−0.00721			
	(0.126)			
Health-care equity×stringency index		−0.000288		
		(0.00621)		
Democracy index×COVID-19 testing policy			0.119	
			(0.829)	
Democracy index×stringency index				0.0232
				(0.0307)
COVID-19 testing policy	0.203		0.151	
	(0.218)		(0.424)	
Stringency index		0.00192		−0.00711
		(0.00861)		(0.0146)
Health-care equity	−0.252	−0.309	−0.270*	−0.348**
	(0.270)	(0.608)	(0.151)	(0.159)
Democracy index	2.365***	2.512***	2.189	0.626
	(0.892)	(0.912)	(1.596)	(2.669)
Per capita GDP (log)	0.419**	0.533***	0.422**	0.547***
	(0.194)	(0.199)	(0.193)	(0.196)
Urban population share	0.00596	0.00332	0.00619	0.00431
	(0.00902)	(0.00932)	(0.00917)	(0.00939)
Population share 65 years old	0.00601	0.0233	0.00696	0.0246
	(0.0350)	(0.0363)	(0.0349)	(0.0353)
Constant	−2.898*	−3.790**	−2.875*	−3.235*
	(1.472)	(1.524)	(1.463)	(1.706)
Estimator	OLS	OLS	OLS	OLS
Regional fixed effects	Yes	Yes	Yes	Yes
No. of countries	126	127	126	127
*R* ^2^	0.665	0.651	0.665	0.652

Standard errors in parentheses. Source: Authors’ compilation based on estimation.

*** *p*<0.01; **p<0.05; *p<0.1.

**Figure 1.**
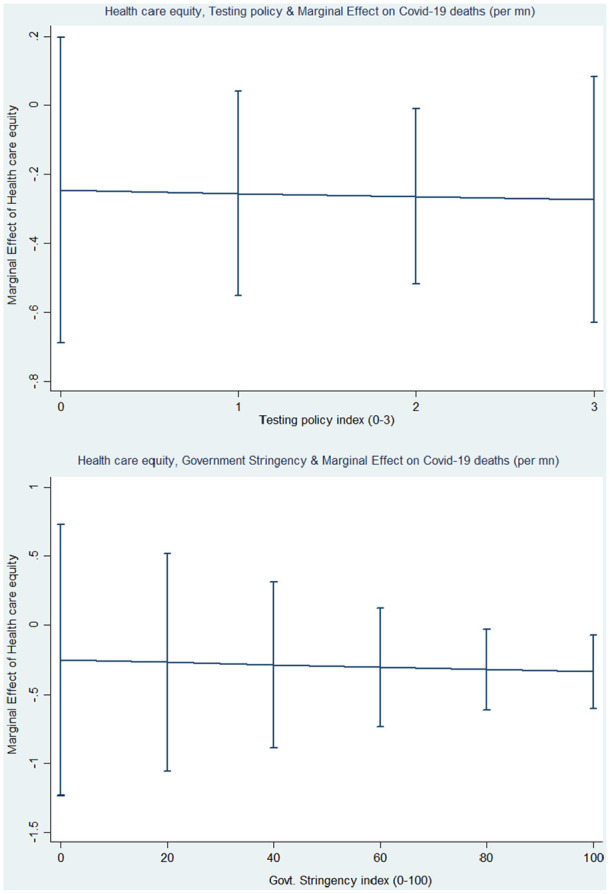
Conditional plots of the marginal effects of health-care equity and government policies on COVID-19 deaths.

As seen in column 1, our interaction term is positive but statistically not different from zero. The health-care equity index on its own (i.e. when the testing policy is 0) has a positive and statistically significant effect on COVID-19 tests per million. However, it is important to note that the interpretation of the interaction terms even in linear models is not so simple. Consequently, a simple *t*-test on the coefficient of the interaction term is not sufficient to examine whether the interaction term is statistically significant [26]. In Figure 1, we display the marginal effect of health-care equity on COVID-19 tests, along the testing policy index on a 0–3 scale.

The graph on the left of Figure 1 shows that health-care equity increases COVID-19 tests per million (log) by 0.62% when the testing policy index is at a maximum score of 3, that is, when a country has an open public testing system in place. This result is significantly different from zero at the 5% level. Regardless, it seems that an equitable health system matters to a far greater extent than the testing policy, suggesting that capacity to carry out testing and act on it is what is critical, not just the policy intentions.

The conditional effect of health-care equity and the stringency index presented in column 2 of Table III show a negative effect. Once again, we resort to the marginal plot to provide a graphical interpretation of the magnitude of the interaction effect. The *y*-axis of the graph on the right (Figure 1) shows that the marginal effect of an additional increase in a unit of the health-care equity index along the stringency index decreases COVID-19 deaths per million (log) when the stringency index is greater than 60 (on a scale of 0–100). The marginal effects are statistically not significant when the stringency index is below 60. For instance, health-care equity reduces COVID-19 deaths per million (log) by 0.30% when government responses to COVID-19 are very strict (i.e. a stringency index of 100), which is statistically significant at the 5% level. Once again, the results suggest that a robust health-care system matters more than the targeted policies, since the effect of an equitable health system on its own has stronger substantive effects. General levels of equity in terms of broad and inclusive governance continue to have the opposite effect independently of all the controls.

**Figure 2.**
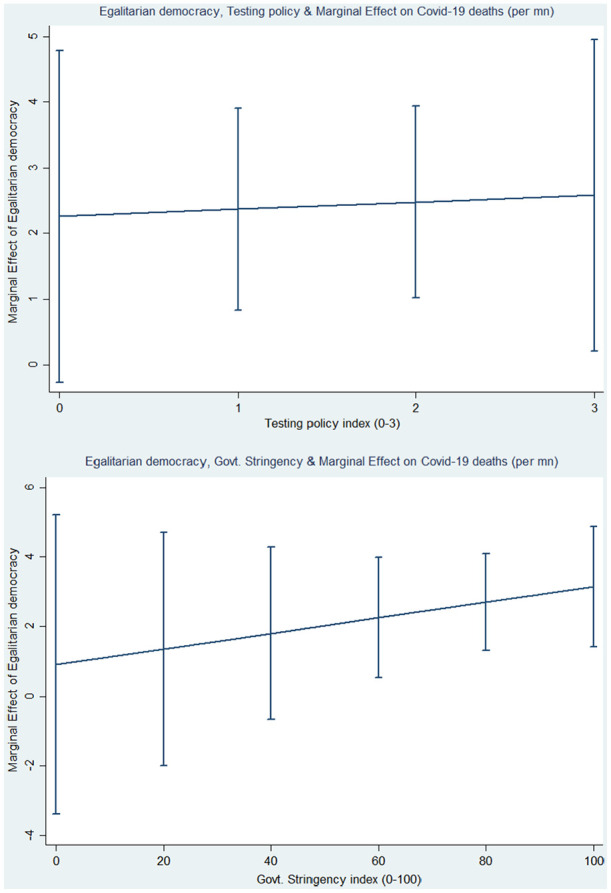
Conditional plots of the marginal effects of egalitarian democracy and government policies on COVID-19 deaths.

In column 3 of Table III, the interaction between egalitarian democracy and testing policy shows a positive effect, but the result is statistically not different from zero. The marginal effect of an additional increase of a unit of egalitarian democracy appears on the *y*-axis of Figure 2 (left graphic), while the stringency index marginal effect is evaluated on the *x*-axis. Figure 2 reveals that egalitarian democracy is conditioned positively on tests, but the effects are not significant along the entire scale. Quite surprisingly, the conditional effect of egalitarian democracy and the stringency index on death are positive. The graphic on the right of Figure 2 reveals that as egalitarian democracy increases in the stringency index above 60, COVID-19 deaths increase. There is thus no additional benefits from broader egalitarian governance processes, even when conditioned by targeted policies. Of course, the targeted policies might also be responses to increasing deaths, which would mean that our conditional effects would be biased. Regardless, taken together, our results suggest strongly that it is an accessible public-health infrastructure that matters for fighting COVID-19, rather than broad egalitarian governance captured in a measure of egalitarian democracy. These results do not support arguments suggesting that policy consensus for fighting a pandemic is easier, or that health outcomes are fairer, when social capital and trust gained through broad egalitarian governance are obtained.

## Conclusions

There seems to be a large body of literature in public health blaming neoliberal epidemics for damaging health outcomes – arguments that have resurfaced following the COVID-19 outbreak [4,9]. Mortality from epidemics is blamed on inequitable governance, where inequities hinder societal cooperation required for achieving collective goods. While equity and welfare should be societal goods pursued for their intrinsic value, how have egalitarian systems of inclusivity and equity broadly helped against the COVID-19 pandemic? Like many others, we find that greater equity in terms of access to health care has mattered for reducing the societal impact of COVID-19, but the mechanism is most likely based on factors associated with health-care system capacity rather than the broad societal impact of egalitarian governance. We find that broad egalitarian societal processes outside the health-care sector have increased deaths from COVID-19, perhaps due to the competing pressures associated with balancing the fight against the virus with economic and political demands from competing interests. Fighting deadly diseases that require extraordinary measures entails more than just societal resources – namely, a clear and targeted physical infrastructure geared towards reaching and treating people. Relying too heavily on societal processes associated with trust and collective action for cauterising the spread of a deadly virus might be a mistake – a hard lesson that countries such as Sweden seem to be realising quite late [11].

Our results support others that suggest that building an equitable health system increases capacity for fighting disease. In a study of the USA, Williams and Cooper [27] argue that COVID-19 has served as a ‘magnifying glass’ that has called attention to the larger issue of health disparities. They note the need for the USAto focus on developing a new ‘herd immunity’ by increasing the resistance of the poor to the spread of disease. Berkowitz, Cené and Chatterjee [28] voice similar concerns, stating that the patterns of power, privilege and inequality in US life are once again observed through this health crisis. The same concerns are raised by Wang and Tang [29] who note that in the case of China, health equity should be the focus of all policies designed to strengthen the country’s health system and emergency responses during health crises in the future. Okoi and Bwawa [30] similarly highlight the difficulties faced by Sub-Saharan African countries in dealing with the COVID-19 outbreak in the absence of basic hygiene facilities. Future studies might examine why some democracies have managed to put in place more targeted policies over others, and identify the precise policies and processes that have affected the disparities in the death rates. Our results suggest that broad egalitarian processes are goods in their own right, but in terms of fighting a deadly disease, targeted health-system capacity building seems like the better bet.

## Supplemental Material

sj-pdf-1-sjp-10.1177_1403494820982106 – Supplemental material for Health-system equity, egalitarian democracy and COVID-19 outcomes: An empirical analysis Click here for additional data file. Supplemental material, sj-pdf-1-sjp-10.1177_1403494820982106 for Health-system equity, egalitarian democracy and COVID-19 outcomes: An empirical analysis by Krishna Chaitanya Vadlamannati, Arusha Cooray and Indra de Soysa in Scandinavian Journal of Public Health

sj-xlsx-1-sjp-10.1177_1403494820982106 – Supplemental material for Health-system equity, egalitarian democracy and COVID-19 outcomes: An empirical analysis Click here for additional data file. Supplemental material, sj-xlsx-1-sjp-10.1177_1403494820982106 for Health-system equity, egalitarian democracy and COVID-19 outcomes: An empirical analysis by Krishna Chaitanya Vadlamannati, Arusha Cooray and Indra de Soysa in Scandinavian Journal of Public Health

## Notes

1. An egalitarian democracy is one in which individuals from all social groups are equally capable of exercising their political rights and freedoms, have little disparities in terms of rights and power resources and where most people are capable of meaningfully influencing political and governing processes. See the Varieties of Democracy project’s website https://www.v-dem.net/en/ (accessed July 2020).

2. The figures were obtained on 20 September 2020 from the World Health Organization [31] (https://covid19.who.int/table).

3. This includes medical staff, medication, hospitals, intensive care units, hospital beds and other necessary infrastructure.

4. Access to health care is defined according to the V-Dem project as adequate health care for the poor that is comparable with the health care accessed by the rich. This variable is explained in greater detail in the Methods.

5. The Worldometer COVID-19 data are available at: https://www.worldometers.info/coronavirus/about/ (accessed June 2020).

6. See https://www.bsg.ox.ac.uk/research/research-projects/coronavirus-government-response-tracker

7. The Global Burden of Disease data can be accessed at: www.healthdata.org/research-article/healthcare-access-and-quality-index-based-mortality-causes-amenable-personal-health (accessed July 2020). The World Bank’s WDI online database can be accessed at: https://databank.worldbank.org/data/reports.aspx?source=world-development-indicators&preview=on# (accessed July 2020).
