# Supplementary material for: Health-system equity, egalitarian democracy and COVID-19 outcomes: An empirical analysis
Source: Scand J Public Health. 2021 Jan 9;49(1):104–13. doi: 10.1177/1403494820982106 (PMC7797351; doi:10.1177/1403494820982106)
Supplement: sj-pdf-1-sjp-10.1177_1403494820982106 – Supplemental material for Health-system equity, egalitarian democracy and COVID-19 outcomes: An empirical analysis [file sj-pdf-1-sjp-10.1177_1403494820982106.pdf]

Appendix Table A4: Data sources and Definitions

Variables	Data definition and sources
Covid-19 tests and deaths per million (log)	Number of Covid-19 tests and deaths per million (log) recorded for country ***c*** as on 25th May 2020 by The Worldometer COVID-19 Data, sourced from https://www.worldometers.info/coronavirus/#countries
Health equity index	VDEM health equality index measures high quality basic health guaranteed to all, sufficient to enable them to exercise their basic rights as adult citizens. The index ranges from -3 to +3, wherein higher value capture basic health is equal in quality and less than five percent (%) of citizens receive low-quality health that probably undermines their ability to exercise their basic rights as adult citizens . We use Five-year average of this index for 2014-2018 years.
Per capita GDP (log)	Five-year average of GDP per capita (log) for 2014-2018 years measured in US$ 2010 constant prices sourced from the World Development Indicators 2019, World Bank.
Urbanization	Five-year average of percentage share of urban population for 2014-2018 years sourced from the World Development Indicators 2019, World Bank.
Democracy index	VDEM´s egalitarian democracy index includes several indicators capturing equal access to power, political resources, liberties and political inclusion, plus the degree of electoral democracy, or polyarchy, indicated by free and fair elections without coercion or violence in a competitive processes. The index is coded on 0-1 scale wherein higher value denote higher egalitarian democratic processes and we use five-year average of this index for 2014-2018 years.
Covid-19 Testing policy index	Testing policy index is coded on the scale of 0-3, wherein 0 suggests there is no adequate Covid-19 testing policy in place, while 3 indicates an open public testing policy in which Covid testing is made available to asymptomatic people by government. The index is developed by Hale et al. (2020) of OxCGRT.
Stringency index	Stringency index is coded on the scale of 0-100, wherein a higher score indicates more stringent government responses to Covid-19. The index is created by Hale et al. (2020) of OxCGRT based on the ordinal values of government policy response on seven variables namely, restrictions of mass gathering, workplace closures, cancellation of public events, public information campaigns, school closures, internal moment restrictions, international travel controls. The index is the average of these seven scores.
